# Density of cigarette retailers near schools and sales to minors in Banyuwangi, Indonesia: A GIS mapping

**DOI:** 10.18332/tid/115798

**Published:** 2020-01-23

**Authors:** Desak M.S.K. Dewi, Susy K. Sebayang, Syifa’ul Lailiyah

**Affiliations:** 1 Research Group for Health and Well-being of Women and Children, Department of Biostatistics and Population Studies, Faculty of Public Health, Universitas Airlangga, Banyuwangi Campus, East Java, Indonesia; 2 Department of Health Policy and Administration, Faculty of Public Health, Universitas Airlangga, Banyuwangi Campus, East Java, Indonesia

**Keywords:** cigarette retailer, density, adolescent, TAPS ban, sales to minors

## Abstract

**INTRODUCTION:**

There are weak regulations and controls on tobacco sales to adolescents in Indonesia, and these may have contributed to the increase in smoking prevalence among adolescents in the country. Our study aims to calculate the density of cigarette retailers near schools and ascertain the factors associated with sales to minors.

**METHODS:**

We conducted geographical mapping by recording the GPS position of cigarette retailers in 15 locations in Banyuwangi District, Indonesia, to assess the density and proximity of cigarette retailers to schools. We interviewed randomly selected retailers, from the geographical mapping, for information on sales to minors, the cheapest price cigarettes are sold and the most popular cigarette brand purchased by adolescents, as well as owners/keepers knowledge of the regulation regarding sales to minors.

**RESULTS:**

We identified 770 retailers of consumer goods in the study location; 28.1% (216) sold cigarettes, with mean density of 1.1 cigarette retailers per 100 m. Of the cigarette retailers, 6.9% were located <25 m from schools and all schools had at least one retailer within a 250 m radius. Owners/keepers of 107 cigarette retailers agreed to be interviewed for information on sales to minors. Brands from Gudang Garam were the most popular among adolescents and the brand from Bentoel, part of British American Tobacco, was the cheapest. The median of the cheapest price sold was US$0.7 per pack. Only 43.6% of retailers ever refused to sell cigarettes to adolescents. Within a school complex, retailers’ refusal to sell cigarettes to adolescents was higher than in other locations.

**CONCLUSIONS:**

Schools in Banyuwangi are surrounded by cigarette retailers. Half of the retailers sell cigarettes at a price affordable by adolescents, attracting adolescents to initiate smoking. There needs to be strict regulation to control cigarette sales to minors, through zoning and licensing in Indonesia.

## INTRODUCTION

Tobacco control regulation in Indonesia is rudimentary, and Indonesia is the only Asian country that has not signed or ratified the WHO Framework Convention on Tobacco Control. Despite the government’s target to reduce adolescent smoking by 5.4% in 2019^1^, the prevalence of adolescent smokers aged 10–18 years in Indonesia continues to increase, from 7.2% in 2013 to 8.8% in 2016, and 9.1% in 2018^1^. A school-based survey reported that a staggering 78.5% of students in Indonesia had ever smoked cigarettes, of which 17.3% tried cigarettes for the first time at an age ≤13 years, and even 5% of male students at an age ≤7 years^2^.

Adolescent smokers have an increased risk of respiratory and non-respiratory diseases, nicotine addiction, and drug use^3^. The long-term impact is reinforced by the fact that most adolescents who smoke will continue to do so regularly throughout adulthood thus increasing the risk for chronic diseases^4^ including heart disease^5^, stroke^6^ and lung cancer^7,8^. Moreover, smoking behaviour in adolescence is also associated with other behaviour problems such as fighting and engaging in unprotected sex^9^, and substance abuse including alcohol, marijuana, and cocaine^3,7,8^.

A high density of cigarette outlets, high exposure to tobacco advertisements at point-of-sale, and open display of cigarette packs in outlets, stimulate tobacco use among adolescents^10-13^. Tobacco retailer density is significantly associated with the prevalence of ever smoking among students^11^ and higher odds of adolescent smoking initiation^14^. Moreover, the frequency of cigarette retailers passed on the way to school is a factor influencing lifetime smoking behaviour^15^. Other studies also reveal that the closer the distance to cigarette retailers, the higher the chance of smoking^16^, the lower the chance of quitting smoking^17^, and higher youth past-30-days smoking frequency^18^.

Tobacco advertisements placed in retailers near schools potentially trigger adolescent smoking behaviour. Tobacco advertising is very creative, appeals to youth^19^ and is associated with greater odds of smoking susceptibility^20^. A study in Germany identified an association between cigarette advertising exposure and having ever tried smoking in school children aged 10–17 years^21^. In Indonesia, 32.4% of high school students found that at least 1 in 15 cigarette advertisements shown to them encouraged them to smoke^22^. Systematic reviews and metaanalysis conclude that exposure to tobacco product displays at the point-of-sale increases both smoking and smoking susceptibility^23,24^.

Several regulations have been issued in Indonesia in an effort to curb smoking among adolescents. The government issued Government Act No 109/2012 prohibiting tobacco sales to children under the age of 18 years, and restricting the design and placement of outdoor tobacco advertisements^25^. The Indonesian Ministry of Education issued the Decree 64/2015 ruling schools as non-tobacco areas^26^, prohibiting smoking, tobacco sales and advertising in schools. At the local level, Banyuwangi regent passed Regulation No. 32/2016 prohibiting the placement of advertisements less than 25 m from schools^27^. However, these regulations do not protect children when they walk outside schools. Other than the prohibition of sales to minors, which is very weakly enforced, there is currently no other regulation controlling cigarette retailers and advertisement placement by retailers. Thus, cigarette retailers with and without advertisements are omnipresent and unregulated. Coupled with the cheap price of cigarettes in Indonesia, the lack of efforts to control cigarette retailers and point-of-sale advertisement near schools means that Indonesian adolescents can easily be drawn to purchase cigarettes near their schools.

There is a scarcity of evidence from Indonesia showing the density of tobacco retailers and points-ofsale. There is only one study conducted in Denpasar, Bali, which found 96.8% of schools have a point-ofsale within a 250 m radius^28^. More studies are needed to provide evidence to support a policy to control cigarette sales to adolescents. Therefore, this study aims to calculate the density of cigarette retailers near schools and the prevalence of factors associated with sales to minors in Banyuwangi, Indonesia. In line with the Indonesian requirements, permits for the study were granted by the local government, while the Ethics Committee of the Faculty of Public Health of Universitas Airlangga approved the study.

## METHODS

### Study design

In 2017 we evaluated Banyuwangi Regent’s Regulation on tobacco advertising ban in the district. Banyuwangi is located in East Java province, the largest tobacco-producing province in Indonesia, and has received many awards for its program and policy innovation, including the health sectors. The evaluation study was conducted on 13 roads and two non road locations where tobacco advertising has been banned in the Banyuwangi subdistrict of Banyuwangi, Indonesia^27^. We expect that if the government of Banyuwangi District is to regulate cigarette retailers, it would come into effect first in these locations. We conducted a geographical mapping of outdoor tobacco advertisement and cigarette retailers in these locations and interviewed owners/keepers of randomly selected retailers from the mapping between November and December 2017. Results of the tobacco advertisement assessment have been previously published^29^. The current study only analysed cigarette retailers. The study was conducted in two phases: Geographical (Geographical Information System – GIS) mapping and a survey of retail owners/keepers.

### Phase 1: Geographical mapping

#### Sampling

To calculate the number of tobacco retailers in the study locations, all types of retailers in the study sites and their GPS locations were recorded. Retailers were defined as stores selling any consumer goods. We observed and recorded outdoor tobacco advertisements placed on retailer shops that were visible from the street and tobacco packs that were clearly displayed. Retailers were categorized as selling cigarettes when tobacco packs were clearly displayed. Outdoor advertisement was defined as all types of tobacco advertisement placed in the vicinity of the retailer shops and clearly visible from the street. We also collected GPS locations of all schools at the study locations, which were defined as formal schools from elementary to high school level. Data from Phase 1 were used as a sampling frame for Phase 2.

#### Data collection

Five teams of two trained enumerators collected the data using KoboCollect, an Android-based data collection application^30^, which is a part of KoBo Toolbox, a free and open source online data entry tool^31^. After training, all enumerators went through a field test period where their mapping and interview skills were assessed, and the data collection online forms were checked and revised as necessary. Acceptable GPS precision was set to less than 10 m. Quality control officers went to 10% of the data points collected by enumerators to check for errors or missing points.

#### Data Analysis

Descriptive analysis was used to assess the density of cigarette retailers and their proximity to schools. We used STATA 14 to calculate the number of retailers with and without outdoor advertisement. Using Quantum GIS 2.8.1, we calculated the proximity of the cigarette retailers to the nearest school using their GPS locations and categorized distance from schools as <25 m, 25–100 m, 100.1–250 m, 250.1–500 m, and >500 m. The 25 m cut-off was used to evaluate the distance where outdoor tobacco advertisement were prohibited by Banyuwangi Regents’ regulation^29^ while distances of 100, 250 and 500 m were used to facilitate comparisons with other studies conducted in Indonesia and several countries in Asia^28,32,33^.

The density of cigarette retailers was indicated by the number of cigarette retailers per 100 m of road length, while for the sports stadium or the city park the density of cigarette retailers was indicated by the number of retailers per 100 m length of all the streets within and surrounding the area. We also calculated the density of retailers in the school complex. The school complex was an area where a collection of schools (elementary, middle, and high school) were located close to one another. We also counted the number of cigarette retailers among all retailers and the number of cigarette retailers featuring outdoor tobacco advertisements.

### Phase 2: Survey of retail owners/keepers

#### Sampling

We randomly selected for interview 150 of 180 consumer goods stores featuring outdoor tobacco advertisement^29^ using a random selection command in Stata V.14. By selecting a sample from stores featuring outdoor tobacco advertisement instead of those observed with cigarette packs on display, we hoped to include more cigarette retailers that might be concealing their cigarettes. Of the 114 storeowners/storekeepers that consented to an interview (3 stores had the same owners/keepers, 14 stores were closed at the time of the visit, and 19 owners/ keepers declined to take part in an interview), 107 sold cigarettes and thus their data were used in the analysis. With this sample size we covered 49.5% of all tobacco retailers in the study location.

#### Data collection

Enumerators visited the randomly selected retailers and explained the survey to the owners or keepers. After consenting, respondents were asked about the cheapest cigarette brands and selling price, the brand most purchased by adolescents, whether they ever refused any adolescents who attempted to purchase cigarettes in their stores, and their knowledge about government regulation prohibit selling cigarettes to people aged <18 years. Data were collected by ten enumerators using Android-based data collection apps on a mobile device^30^. The average interview time was 22 minutes for each respondent. Respondents who were not available for interview immediately after the consenting process were interviewed later at a more convenient time to the respondents. Another visit took place by a quality control team to 10% randomly-selected interviewed respondents for quality control.

#### Data analysis

Descriptive statistics (frequencies and percentages) were computed using Stata V.14. The cheapest cigarette price was categorized as <US$0.7 or ≥US$0.7 per pack, based on the median of data. We conducted a chi-squared analysis of the association between the lowest cigarette price and knowledge about a regulation banning sales to minors and retailers’ refusal to sell cigarettes to adolescents.

## RESULTS

### Phase 1: Geographical mapping

We identified 770 consumer goods retailers in the study location, of which 28.1% (216) sold cigarettes. Only one street in the 15 locations was free of cigarette retailers (Table 1). The overall mean density of cigarette retailers in 15 locations was 1.1 retailers per 100 m. The location with the highest cigarette retailer density (2.2 retailers/100 m) was on the main road (Jalan R. Wijaya). The density in the city park was 1.6 retailers/100 m, while in the sports stadium it was 0.8 retailers/100 m (Table 1).

**Table 1:** Number and percentage of schools, cigarette retailers, and density, Banyuwangi, East Java, Indonesia, 2017

*Location*	*Schools*		*Stores*		*Stores selling cigarettes*		*Road length (m)*	*Density (per 100m)*
	*n*	*%*	*n*	*%*	*n*	*%*		
Jl. Argopuro	1	3.2	37	4.8	21	56.8	2600	0.8
Jl. R. Wijaya	5	16.1	101	13.1	33	32.7	1500	2.2
Jl. M.H. Thamrin	2	6.5	109	14.2	13	11.9	1900	0.7
Jl. Hayam Wuruk	1	3.2	27	3.5	11	40.7	700	1.6
Jl. Widuri	1	3.2	59	7.7	19	32.2	1000	1.9
Jl. Mawar	2	6.5	18	2.3	10	55.6	900	1.1
Jl. HOS Cokroaminoto (school complex)	4	12.9	64	8.3	12	18.8	850	1.4
Jl. Wijaya Kusuma (school complex)	5	16.1	5	0.6	3	60.0	850	0.4
Jl. Gajah Mada	0	0.0	22	2.9	4	18.2	1000	0.4
Jl. Brawijaya	1	3.2	93	12.1	33	35.5	3300	1.0
Jl. S. Parman	2	6.5	111	14.4	25	22.5	2000	1.3
Jl. Simpang Gajah Mada	1	3.2	2	0.3	0	0.0	240	0.0
Jl. Ahmad Yani	5	16.1	67	8.7	12	17.9	1200	1.0
GOR Tawang Alun (sports stadium)	0	0.0	11	1.4	10	90.9	1230	0.8
Taman Sri Tanjung (city park)	1	3.2	44	5.7	10	22.7	610	1.6
**Total**	**31**	**100.0**	**770**	**100.0**	**216**			**1.1**

**Figure 1:**
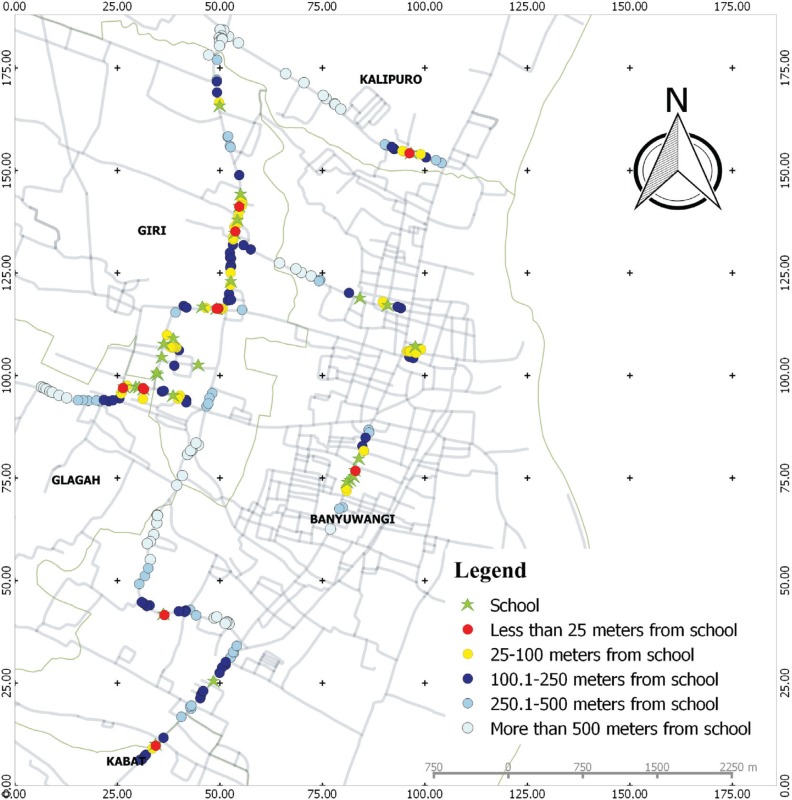
Density and proximity of cigarette retailers near schools in Banyuwangi, East Java, Indonesia

There were 31 schools in the study locations. Using their GPS locations, we found that 6.9% of the 216 cigarette retailers were located <25 m from schools, and most of the retailers (27.8%) were located at a distance of 100.1–250 m from schools. The closest cigarette retailers from a school were only 1.7 m away. We found that 9 (29.0%) of the 31 schools in the study sites had at least one cigarette retailer within 25 m, 25 (80.6%) schools within 100 m, and all schools had at least one cigarette retailer within a 250 m radius (Figure 1).

In all, 73.1% of the retailers featured outdoor tobacco advertisements. Of retailers located <25 m, 66.7% had outdoor tobacco advertisements placed in their stores, which was less than those located farther from schools (Table 2). The most prevalent types of advertisements near schools were posters and banners, but banners were more prevalent within <25 m (Table 2).

**Table 2:** Number and percentage of cigarette retailers with outdoor tobacco advertisement (n=158 ) and advertisement type (n=158) by distance from the nearest schools, Banyuwangi, East Java, Indonesia, 2017

	*<25 m (n=15)*		*25–100 m (n=49)*		*100.1–250 m (n=60)*		*250.1–500 m (n=42)*		*>500 m (n=50)*		*Total (n=216)*	
	*n*	*%*	*n*	*%*	*n*	*%*	*n*	*%*	*n*	*%*	*n*	*%*
**Number of cigarette retailers with advertisement**	10	66.7	36	73.5	43	71.7	34	81.0	35	70.0	158	73.1
**Advertisement type**												
Poster	4	40.0	16	44.4	15	34.9	11	32.4	11	31.4	57	36.1
Banner	6	60.0	9	25.0	13	30.2	10	29.4	11	31.4	49	31.0
Small billboard (0.3×0.6 m)	0	0.0	1	2.8	5	11.6	5	14.7	1	2.9	12	7.6
Medium billboard (0.3×0.6 m –1.2×1.2 m)	0	0.0	1	2.8	0	0.0	0	0.0	2	5.7	3	1.9
Shop/stall sign	0	0.0	0	0.0	2	4.7	2	5.9	1	2.9	5	3.2
Stickers	0	0.0	9	25.0	8	18.6	6	17.6	9	25.7	32	20.3

### Phase 2: Survey of retail owners/keepers

Of the 107 interviewed cigarette retail owners/keepers, 101 provided information on the cheapest cigarette price they sold, 94 provided information on refusal to sell cigarettes to adolescents, 92 provided information on the popular cigarette brand among adolescent, 101 provided information on the cheapest cigarette brand, and 107 provided information on knowledge of the regulation prohibiting cigarette sales to minors.

The cheapest cigarette price sold ranged from US$0.4 to US$1.5 per pack (exchange rate: US$ 1 = IDR 14080). Of all cigarette retailers, 48.0% sold the cheapest pack of cigarettes at <US$0.7. We found that 43.6% of the respondents stated that they had at least once refused to sell cigarettes to adolescents. When we categorized those selling the cheapest pack of cigarettes at <US$0.7 per pack versus ≥US$0.7 per pack, 56.1% of cigarette retailers selling at the lowest price of <US$0.7 per pack reported to have had refused once to sell cigarettes to adolescents, while only 43.9% of cigarette retailers with the lowest price of ≥US$0.7 per pack had ever refused to sell cigarettes to adolescents. However, the difference was not statistically significant (p=0.39). In the school complex, 90.0% of retailers had once refused to sell cigarettes to minors. This number was higher than that at the sports stadium where only 20.0% of retailers had ever refused adolescents attempting to purchase cigarettes. There were only 38 (35.5%) of cigarette retailer owners/keepers who knew about the regulation prohibiting cigarette sales to minors. There was no significant association between retailers’ refusal to sell cigarettes to adolescents and their knowledge about the regulation that prohibited cigarette sales to minors (p=0.07).

**Figure 2:**
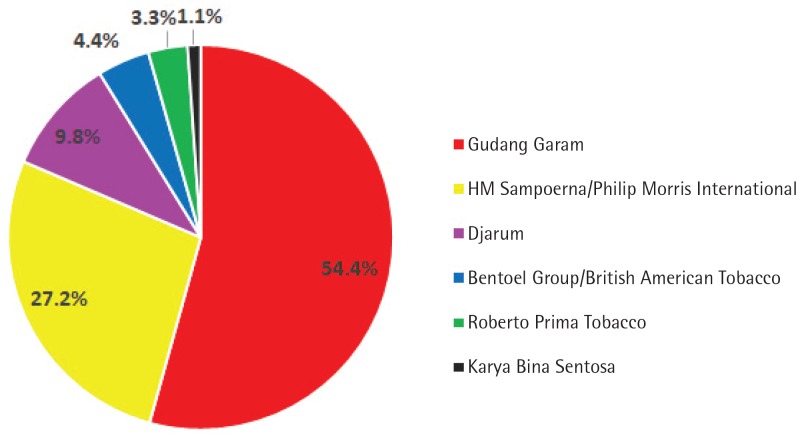
Distribution of the most popular cigarettes among adolescents in Banyuwangi, East Java, Indonesia, by tobacco company

**Figure 3:**
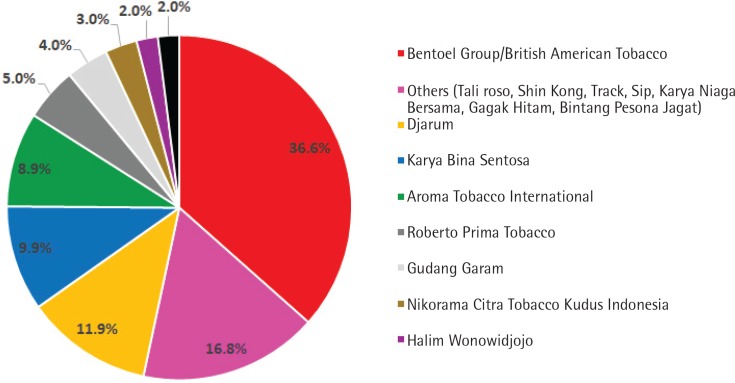
Distribution of the cheapest cigarettes in Banyuwangi, East Java, Indonesia, by tobacco company

The most popular cigarettes among children were from Gudang Garam (54.4%), which included brands such as Surya (50.0%), Gudang Garam (3.3%), and Pro-Mild (1.1%). The second most popular cigarettes were from HM Sampoerna/Philip Morris International (27.2%) (Figure 2). The cheapest cigarettes sold were from Bentoel Group/British American Tobacco (36.6%), which included brands such as Tali Jagat (28.7%), Lucky Strike (6.9%), and Dunhill (1.0%) (Figure 3). The cheapest cigarette was Tali Jagat, which was sold for 0.4–0.9 US$ per pack.

## DISCUSSION

### Main findings

Our study shows that the mean density of cigarette retailers in Banyuwangi was 1.1 retailers per 100 m (range: 0.4–2.2 per 100 m). Most of the retailers (27.8%) were located at a distance of 100.1–250 m from schools and had outdoor tobacco advertisements (73.1%). Of all retailers, only 43.6% had once refused to sell cigarettes to adolescents. The most popular cigarettes for adolescents were of Gudang Garam Brands (50.0%), and the cheapest cigarettes were from Bentoel Group, which is a part of British American Tobacco (36.6%). The cheapest brand was Tali Jagat, which was sold at 0.4–0.9 US$ per pack.

All of the schools in our studies had neighbouring tobacco retailers within 250 m, which was higher than that reported in Bali, Indonesia, where 96.8% of schools had a tobacco retailer within a 250 m radius^28^. The nearest cigarette retailers to schools in this study (1.7 m) were also closer than reported for Bali (2.9 m)^28^.

We have previously reported that 23.4% of all types of retailers placed outdoor tobacco advertisements on their stores^29^. The current study shows that a much higher percentage (73.1%) of cigarette retailers featured outdoor tobacco advertisements. Comparing our results on outdoor tobacco advertisement with that from other countries is difficult as there are currently not many other countries that allow outdoor tobacco advertisements.

The price of cigarettes in our study was lower than that in Malaysia, which sets the lowest price of cigarettes at US$1.7^34^. Our finding is in line with the fact that Indonesia occupies the 9th position of 88 countries with the lowest cigarette prices globally and is 6th out of 29 Asian countries with the lowest cigarette prices^35^.

The high density of cigarette retailers in the study location was not surprising as there were very limited regulations governing cigarette retailers and tobacco sales to minors in Indonesia. The high refusals of purchase attempts in the school complex may indicate higher compliance to the regulation banning tobacco sales to people aged <18 years. However, our results show that retailers’ knowledge of the regulation was not associated with selling tobacco to minors. This indicates that the refusal number perhaps was more of a reflection of adolescents’ attempt to purchase cigarettes near their schools.

Although there were violations to the advertisement ban within 25 m of schools, the lower percentage of retailers posting tobacco advertisement <25 m from schools compared to retailers located ≥25 m might indicate some positive results from the implementation of the ban. Nevertheless, the number of violations still indicates weak enforcement of the regulation.

This study also found that cigarettes were sold at very affordable prices (0.4–1.5 US$ per pack). This price is much lower than the pocket money of many Indonesian children, reported to be on average US$39.1 per month or around US$1.3 per day^36^. Our study found that Surya from Gudang Garam was the most popular brand among adolescents. This particular brand has a variety of packaging, including a can of 50 cigarette sticks, which can be sold individually. This allows adolescents to purchase a cigarette at a fraction of the cost of a pack. Also, it might not be a coincidence that Gudang Garam was also the company with the highest shares of outdoor tobacco advertisement^29^. The combination of the high density of retailers close to schools, the high number of tobacco retailers featuring outdoor tobacco advertisements, the affordable price and the single stick sales, if maintained, will continue to increase the smoking prevalence among adolescents in Indonesia.

### Policy implications

Although the Indonesian Ministry of Education has ruled out cigarette sales in schools as part of the no-smoking area regulation, adolescents can attempt to purchase cigarettes outside of their schools. The availability of cigarette retailers near schools and even <25 m from schools facilitates adolescents to initiate smoking. Although there were violations to the advertisement ban within 25 m of schools, the lower number of retailers placing outdoor tobacco advertisements within 25 m of schools in Banyuwangi shows that the regulation is a potentially effective measure. This means that the total ban on outdoor tobacco advertisement should be pursued in this District.

This study and the study in Bali show that the Indonesian government needs to regulate tobacco sales by establishing zones where tobacco sales are permitted. Ban of tobacco sales within 100 yards (91.44 m) of educational institutions has been shown to reduce retailer density in India^37^. The study in Bali suggests prohibiting sales within a 500 m radius may deliver the greatest impact and the adoption of tobacco retailer ban within at least 100 m radius from schools will likely reduce youth exposure to cigarette marketing^28^. As many adolescents attempt to purchase, regulation for cigarette trader licensing is also worth considering. The licensing can enforce the prohibition of sales to minors as licensed retailers will be required to ask for identity cards for proof of age and must refuse to sell cigarettes to those who cannot show their identity card^28,38^. All these regulations, however, will not be effective without proper surveillance and heavy penalties. A study reported that high surveillance and heavy penalties for violation are an important aspect to ensure reduction in smoking among adolescents^14^. It has been shown in Taiwan that a heavy penalty for selling to minors deters cigarette retailers from sales to minors^28,38^. Tobacco control regulation in Indonesia is rudimentary but even the currently available regulation does not constitute a strong measure against violations and the surveillance system to detect violations needs upgrading^29^. Retailer zones can be easily implemented in Banyuwangi, as this District has previously successfully placed a moratorium for franchised mini-markets to protect small scale businesses. Although most cigarette retailers in the District are small scale businesses, the District’s focus on ecotourism development^29^ can be a means to stimulate small scale business to sell items that are more in line with ecotourism.

The government of Indonesia still allows the use of terms that may insinuate a safer cigarette product such as ‘light’ and ‘mild’ as part of cigarette brand names. Cigarette with flavours that are appealing to adolescents such as fruits and mints, have recently become more popular. Our study, however, did not assess such word descriptors in the cigarette brands. Therefore, further study should include these types of cigarettes to provide evidence for policy change.

### Strengths and limitations

This is the second paper illustrating the density of cigarette retailers in Indonesia and the first conducted in Java, the most populous island in the country. This study was carried out only on main roads where the advertisement ban was already effective; thus, our results might be an underestimation of the actual cigarette retailer density. We assessed advertisements on retailers that were visible from the street instead of the total advertisement inside the stores. Our estimates for advertisement, therefore, are an underestimation of the total efforts of the tobacco industry to persuade purchase through advertisement at point-of-sale. We also did not assess the actual frequency of purchase either in packs or single sticks by adolescents and thus could not provide more detailed information on their purchasing behaviour.

## CONCLUSIONS

Cigarette vendors surround schools in Banyuwangi. Most of the cigarette retailers posted outdoor tobacco advertisements and sold cigarettes at a price affordable by adolescents. All of these may attract adolescents to initiate smoking. There need to be regulations such as zoning and licensing to control cigarette sales to minors.

## References

[1] Kementerian Kesehatan Republik Indonesia, Kementerian Kesehatan Republik Indonesia Badan Penelitian dan Pengembangan Kesehatan (2018). Laporan Nasional RISKESDAS 2018.

[2] Kusumawardani N, Rachmalina S, Wiryawan Y (2015). Perilaku Berisiko Kesehatan pada Pelajar SMP dan SMA di Indonesia, Hasil Survey Nasional Kesehatan Berbasis Sekolah di Indonesia.

[3] World Health Organization Health effects of smoking among young people.

[4] U.S. Department of Health and Human Services (2012). Preventing Tobacco Use Among Youth and Young Adults: A Report of the Surgeon General.

[5] Khan RJ, Stewart CP, Davis SK, Harvey DJ, Leistikow BN (2015). The risk and burden of smoking related heart disease mortality among young people in the United States. Tob Induc Dis.

[6] Shah RS, Cole JW (2010). Smoking and stroke: the more you smoke the more you stroke. Expert Rev Cardiovasc Ther.

[7] Department of Health, Australian Government Smoking and tobacco and young people.

[8] Park S (2011). Smoking and Adolescent Health. Korean J Pediatr.

[9] American Cancer Society Health Risks of Smoking Tobacco.

[10] Henriksen L (2015). The retail environment for tobacco: a barometer of progress towards the endgame. Tob Control.

[11] Adams ML, Jason LA, Pokorny S, Hunt Y (2013). Exploration of the link between tobacco retailers in school neighborhoods and student smoking. J Sch Health.

[12] Lipperman-Kreda S, Grube JW, Friend KB, Mair C (2016). Tobacco outlet density, retailer cigarette sales without ID checks and enforcement of underage tobacco laws: associations with youths’ cigarette smoking and beliefs. Addiction.

[13] McCarthy WJ, Mistry R, Lu Y, Patel M, Zheng H, Dietsch B (2009). Density of tobacco retailers near schools: effects on tobacco use among students. Am J Public Health.

[14] Shortt NK, Tisch C, Pearce J, Richardson EA, Mitchell R (2016). The density of tobacco retailers in home and school environments and relationship with adolescent smoking behaviours in Scotland. Tob Control.

[15] Gwon SH, Yan G, Huang G, Kulbok PA (2017). The influence of tobacco retailers on adolescent smoking: prevention and policy implications. Int Nurs Rev.

[16] Marashi-Pour S, Cretikos M, Lyons C, Rose N, Jalaludin B, Smith J (2015). The association between the density of retail tobacco outlets, individual smoking status, neighbourhood socioeconomic status and school locations in New South Wales, Australia. Spat Spatiotemporal Epidemiol.

[17] CounterTobacco.org Why Retail Tobacco Control is Important.

[18] Lipperman-Kreda S, Mair C, Grube JW, Friend KB, Jackson P, Watson D (2014). Density and proximity of tobacco outlets to homes and schools: relations with youth cigarette smoking. Prev Sci.

[19] Sebayang SK, Rosemary R, Widiatmoko D, Mohamad K, Trisnantoro L (2012). Better to die than to leave a friend behind: industry strategy to reach the young. Tob Control.

[20] Chido-Amajuoyi OG, Mantey DS, Clendennen SL, Pérez A (2017). Association of tobacco advertising, promotion and sponsorship (TAPS) exposure and cigarette use among Nigerian adolescents: implications for current practices, products and policies. BMJ Glob Health.

[21] Hanewinkel R, Isensee B, Sargent JD, Morgenstern M (2010). Cigarette Advertising and Adolescent Smoking. Am J Prev Med.

[22] Prabandari YS, Dewi A (2016). How do Indonesian youth perceive cigarette advertising? A cross-sectional study among Indonesian high school students. Glob Health Action.

[23] Robertson L, McGee R, Marsh L, Hoek J (2015). A systematic review on the impact of point-of-sale tobacco promotion on smoking. Nicotine Tob Res.

[24] Robertson L, Cameron C, McGee R, Marsh L, Hoek J (2016). Point-of-sale tobacco promotion and youth smoking: a meta-analysis. Tob Control.

[25] Presiden Republik Indonesia, Kementerian Hukum dan Hak Asasi Manusia (2012). Peraturan Pemerintah Republik Indonesia Nomor 109 tahun 2012 tentang Pengamanan Bahan yang Mengandung Zat Adiktif berupa Produk Tembakau Bagi Kesehatan.

[26] Kementerian Pendidikan dan Kebudayaan RI, Kementerian Pendidikan dan Kebudayaan (2015). Peraturan Menteri Pendidikan dan Kebudayaan Republik Indonesia Nomor 64 tahun 2015 tentang Kawasan Tanpa Rokok di Lingkungan Sekolah.

[27] Bupati Banyuwangi, Pemerintah Kabupaten Banyuwangi (2016). Peraturan Bupati Banyuwangi Nomor 32 Tahun 2016 tentang Perubahan atas Peraturan Bupati Banyuwangi Nomor 6 tahun 2013 tentang Pedoman Pelaksanaan Peraturan Daerah Kabupaten Banyuwangi Nomor 10 tahun 2012 tentang Penyelenggaraan Reklame.

[28] Astuti PAS, Mulyawan KH, Sebayang SK, Kurniasari NMD, Freeman B (2019). Cigarette retailer density around schools and neighbourhoods in Bali, Indonesia: A GIS mapping. Tob Induc Dis.

[29] Sebayang SK, Dewi DMSK, Lailiyah Su, Ahsan A (2018). Mixedmethods evaluation of a ban on tobacco advertising and promotion in Banyuwangi District, Indonesia. Tob Control.

[30] KoboToolBox [program] (2012). Version v1.14.0a.

[31] Harvard Humanitarian Initiative About Kobotoolbox.

[32] Phetphum C, Noosorn N (2019). Tobacco Retailers Near Schools and the Violations of Tobacco Retailing Laws in Thailand. J Public Health Manag Pract.

[33] Balappanavar AY, Mohanty V, Hussain A (2017). Compliance with Tobacco Promotion and Sale Laws in School Neighbourhoods in India. Asian Pac J Cancer Prev.

[34] Liber AC, Ross H, Omar M, Chaloupka FJ (2015). The impact of the Malaysian minimum cigarette price law: findings from the ITC Malaysia Survey. Tob Control.

[35] NUMBEO Price Rankings by Country of Cigarettes 20 Pack (Marlboro) (Markets).

[36] Markustianto D Mendidik Anak Sekolah dari Uang Saku.

[37] Mistry R, Pednekar M, Pimple S (2015). Banning tobacco sales and advertisements near educational institutions may reduce students’ tobacco use risk: evidence from Mumbai, India. Tob Control.

[38] Ackerman A, Etow A, Bartel S, Ribisl KM (2017). Reducing the Density and Number of Tobacco Retailers: Policy Solutions and Legal Issues. Nicotine Tob Res.

